# Focused assessment with sonography for HIV-associated tuberculosis (FASH): a short protocol and a pictorial review

**DOI:** 10.1186/2036-7902-4-21

**Published:** 2012-11-21

**Authors:** Tom Heller, Claudia Wallrauch, Sam Goblirsch, Enrico Brunetti

**Affiliations:** 1 Department of Internal Medicine, Infectious Diseases, Klinikum Muenchen-Perlach, chmidbauerstr. 44, Munich, 81737, Germany; 2 , Saint Paul, MN, 55105, USA; 3 Department of Infectious Diseases, San Matteo Hospital Foundation, University of Pavia, Via Taramelli 5, Pavia, 27100, Italy

**Keywords:** HIV, TB, Co-infection, Ultrasound, Focused assessment, Resource-limited setting.

## Abstract

**Background:**

Ultrasound can rapidly identify abnormal signs, which in high prevalence settings, are highly suggestive of extra-pulmonary tuberculosis (EPTB). Unfortunately experienced sonographers are often scarce in these settings.

**Methods:**

A protocol for focused assessment with sonography for HIV-associated tuberculosis (FASH) which can be used by physicians who are relatively inexperienced in ultrasound was developed.

**Results:**

The technique as well as normal and pathological findings are described and the diagnostic and possible therapeutic reasoning explained. The protocol is intended for settings where the prevalence of HIV/TB co-infected patients is high.

**Conclusion:**

FASH is suitable for more rapid identification of EPTB even at the peripheral hospital level where other imaging modalities are scarce and most of the HIV and TB care will be delivered in the future.

## Background

In sub-Saharan Africa, the convergence of human immunodeficiency virus (HIV) and tuberculosis (TB) epidemics has led to a resurgence of extrapulmonary TB (EPTB). EPTB accounts for 15% to 20% of all TB cases reported by TB control programs in the African region [1]. Diagnosis is hampered by the difficulty to obtain material, poor sensitivity of microscopy, and the limited availability of culture techniques. Consequently, the diagnosis is usually based on clinical case definitions [2].

Common manifestations of EPTB include pericardial effusion, pleural effusion, and abdominal TB. Tuberculous pericarditis is consistently reported as the predominant cause of pericardial effusion in Southern Africa and is in most of the cases associated with HIV co-infection [3]. Cardiac tamponade is the most severe clinical presentation of TB pericarditis and constitutes a life threatening event that requires immediate medical intervention [4]. Pleural effusion, especially when unilateral and associated with HIV infection, is most likely to be caused by tuberculosis in countries with a high TB incidence [5]. Typical ultrasound (US) findings of abdominal TB include retroperitoneal and mesenteric lymphadenopathy with node diameter greater than 1.5 cm, multiple splenic hypoechoic nodules between 0.5 and 1 cm, and patterns of ascites [6].

Ultrasound (US) can rapidly identify abnormal signs, which in high prevalence settings, will be highly suggestive of EPTB [7,8], but trained sonographers are scarce in resource-limited settings to detect these findings. In recent years, the introduction of point-of-care US performed by clinicians has been successfully implemented in a variety of settings. Specific protocols need to be provided for questions applicable in different settings and patient populations. Short protocols have been successfully established in emergency medicine such as focused assessment with sonography for trauma [9] for trauma victims. In a similar approach, a focused assessment with sonography for HIV-associated TB (FASH) protocol was developed, modeled on these protocols and experiences in emergency medicine [10].

## Methods

A list of key ultrasound findings was included in the protocol. The selection criteria were based on the relative ease to recognize the findings and the relevance with respect to diagnostic and therapeutic decisions. The FASH basic assessment (Table 1) is meant to identify pathological effusions in the body cavities. In the appropriate clinical setting, these are highly suggestive of EPTB. The FASH plus looks for other findings like enlarged lymph nodes and micro-abscesses of the spleen, which are frequently seen in HIV-infected patients and point towards HIV/TB co-infection. These are slightly more difficult to recognize, but the examiner should attempt to visualize the relevant structures as experience increases.

**Table 1:** Clinical questions addressed by FASH

**FASH-basic**	**Clinical questions**	**FASH-plus**	**Clinical questions**
Presence of pericardial effusion?	Pericardial TB?	Presence of periportal/para-aortic lymph nodes?	Abdominal TB?
Presence of pleural effusion?	Pleural TB?	Presence of focal liver lesion?	Liver abscess? TB?
Presence of ascites?	Possibly abdominal TB?	Presence of focal splenic lesions?	Disseminated TB?

## Results and discussion

### Protocol of the exam

#### Patient preparation

Patient is placed in a comfortable supine position. The procedure is briefly explained to the patient reassuring him/her that it is completely painless. A curved 3.5 to 5-MHz transducer is used for the examination of the abdomen. Gain, depth, and focus might need to be adjusted for optimal imaging. Sufficient amount of ultrasound gel is placed on the transducer and the patient's abdomen to ensure good acoustic coupling between the probe and skin. For the beginning of the examination (probe position 1a and 1b), the patient is asked to place his/her arms beside the body to relax the muscles of the abdominal wall.

#### Examination

**Figure 1:**
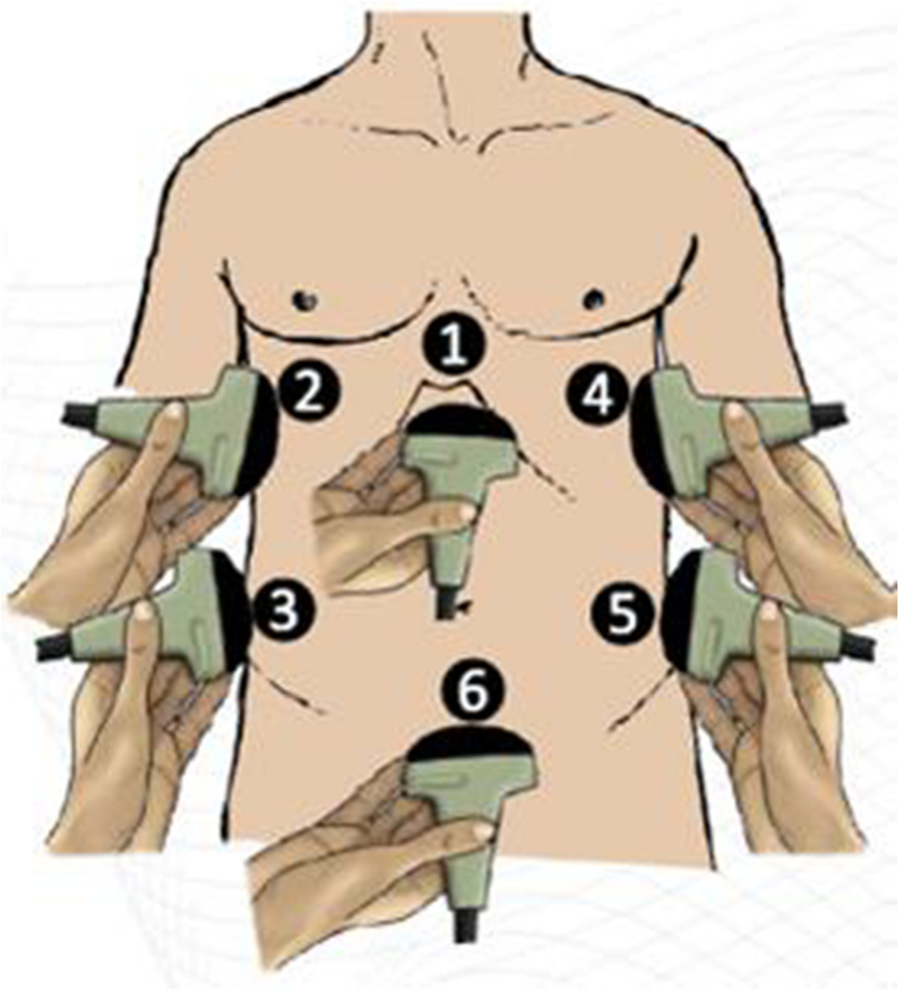
Schematic drawing of the ultrasound probe positions during the FASH examination.

**Figure 2:**
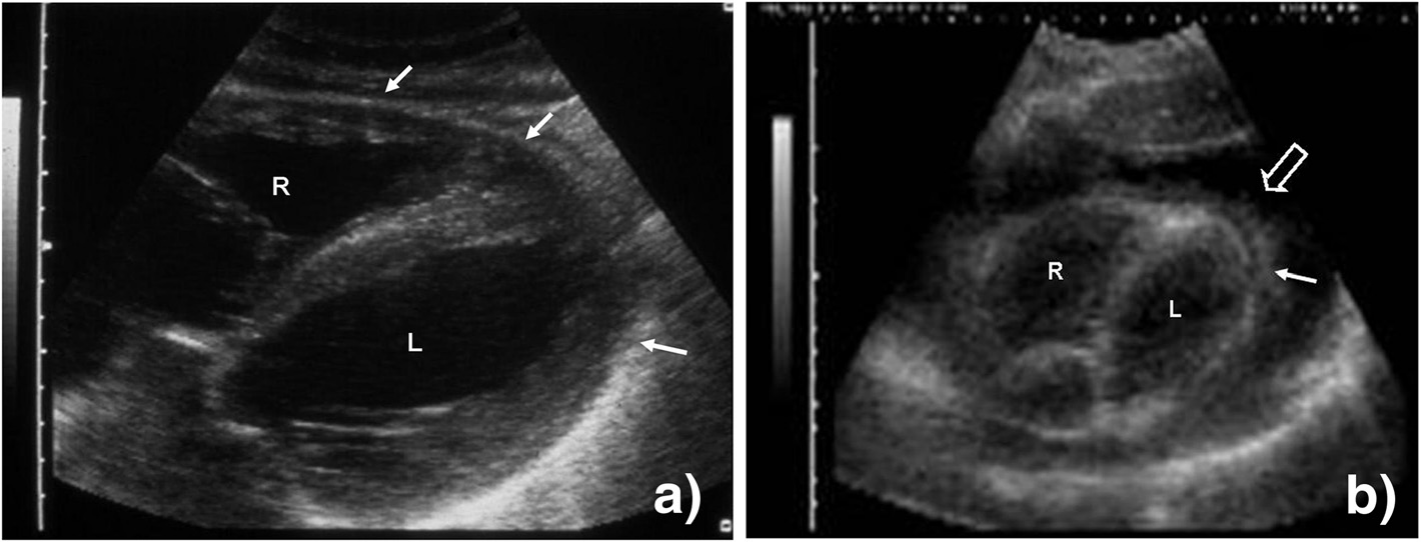
**Probe position 1a.** (**a**) Right (R) and left (L) ventricle of the heart are visible. The pericardium surrounds the heart as an echogenic rim (filled arrow). (**b**) Right (R) and left (L) ventricle of the heart are visible. The heart is surrounded by a large echo-free rim (open arrow), the pericardial effusion. On the visceral side, echogenic fibrinous material (filled arrow) is visible inside the effusion.

The patient is scanned by six different probe positions (Figure 1). In each position, different questions are addressed:

Probe position 1a: pericardial effusion

a. *Indication*: Pericardial effusion should be considered in every HIV-positive individual with signs of breathlessness, retrosternal pain, and signs of progressive heart failure, tachycardia or hypotension. A chest X-ray showing cardiomegaly is frequently caused by pericardial effusion and should be further assessed by ultrasound.

b. *Probe position*: The probe is placed transverse in the epigastric angle; the transducer is then tilted cranially to get a view of the intra-thoracic organs and the heart. The patient should try to relax his abdominal muscles (arms placed beside the body) as an attempt is made to ‘scoop’ the transducer under the ribs. Commands asking the patient to inspire might help to displace the heart caudally and improve visualization.

c. *Normal findings*: The left lobe of the liver will be visible in the upper parts of the image serving as an acoustic window. Below this, the right atrium as well as the right ventricle will be seen and can be easily recognized by the rhythmic contractions. The right ventricle increases in size during diastole and contracts during the systolic phase of the heart action. The pericardium is seen as an echogenic structure between the liver and the heart; the parietal and visceral pericardium are inseparable (Figure 2a). In the distant parts of the US image, the left heart may be seen.

d. *Pathological findings*: Pericardial effusion mainly shows as an anechoic, black rim around the heart separating the visceral and parietal pericardia (Figure 2b). The rim might surround the entire heart and be visible on the side of the left ventricle as well. Frequently, echogenic material like fibrin streaks can be seen floating in the anechoic effusion (Additional file 1: Video 1); occasionally, the whole effusion appears echogenic mainly due to high protein and particle content in purulent exudative effusions. In cases of pericardial tamponade, tachycardia and impaired filling of the right atrium and/or ventricle might be seen. Sonographic changes of tamponade, mainly diastolic collapse of the right ventricle (Additional file 2: Video 2) due to increased pericardial pressure, might in some cases precede clinical symptoms and would then be termed ‘pre-tamponade’.

e. *Interpretation*: An anechoic rim surrounding the heart confirms the presence of a pericardial effusion. In a settings where TB has a high prevalence like in Sub-Saharan Africa, pericardial TB is the most frequent cause of pericardial effusion [11]. A case series from the Western Cape region, South Africa, showed 70% of the pericardial effusions due to TB [12]. In a series from Tanzania, TB was the cause in virtually all HIV co-infected patients [13]. Other differential diagnoses such as malignancies, e.g., lymphoma or Kaposi's sarcoma (KS) should be considered, especially in patients with suggestive lesions in other parts of the body. A thorough examination of the skin, the intra-oral mucosa, and lymph nodes should be done.

We would not recommend diagnostic pericardiocentesis in a hemodynamically stable patient as the risk of iatrogenic injury would be high. If the patient is hemodynamically unstable and the exam suggests cardiac tamponade, ultrasound can be used to guide pericardiocentesis, which might be life saving [4]. Pericardial effusions due to TB are often straw-colored; however, bloody or putrid effusions (raising suspicion of bacterial super-infection) are seen. Blood stained effusions are also suggestive of malignancy like KS.

If pericardial fluid is withdrawn, TB culture should be attempted as AFB smear is almost invariably negative [14]. However, cultures are not helpful for the immediate diagnosis. Rapid DNA amplification techniques allow timely diagnosis; unfortunately, these techniques are often not available in the poor-resource setting. The roll-out of new technologies like the GeneXpert MTB in South Africa is promising as its technology might also be used on non-respiratory samples [15]; whether this will change the diagnostic approach cannot be concluded yet as data on its sensitivity and specificity in pericardial effusions is still lacking.

In an HIV patient with pericardial effusion, anti-TB treatment is warranted, particularly when severe immunosuppression and low CD4 counts are known. Additionally, we would recommend steroid treatment especially in case of large and hemodynamically relevant pericardial effusions [4]. It should be noted that malignant effusions will also respond to steroid treatment, but patients will relapse after steroids are tapered. All HIV-positive patients with TB pericarditis should start anti-retroviral therapy (ART). The exact timing of ART initiation in TB co-infected patients is still unclear; we would start after 2 to 8 weeks of TB treatment.

**Figure 3:**
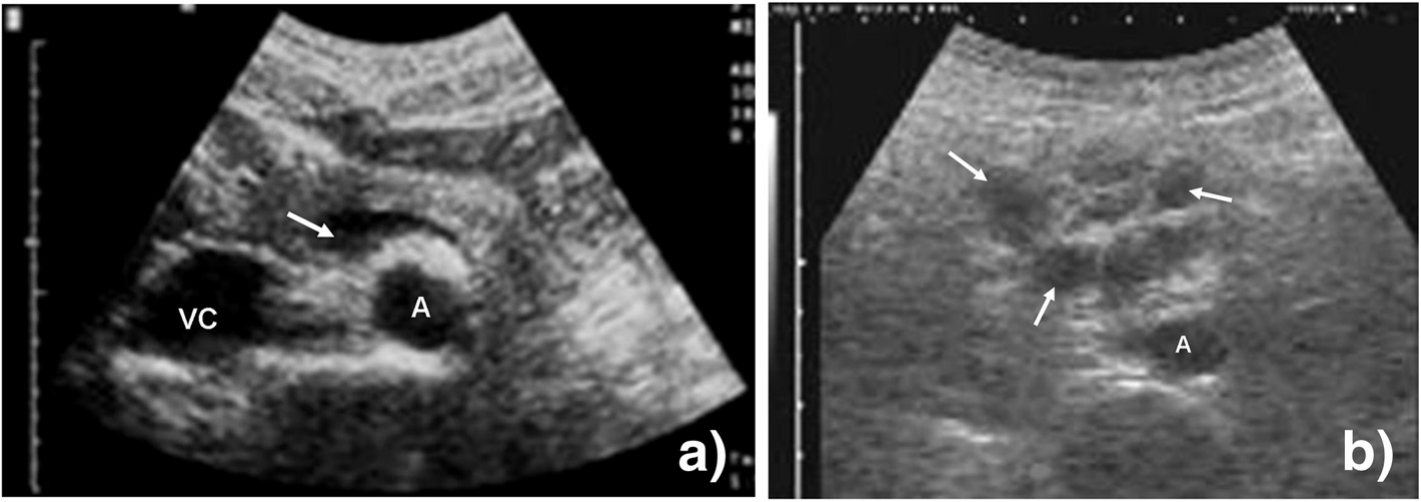
**Probe position 1b.** (**a**) Vascular structures (abdominal aorta (A), inferior vena cava (VC), splenic vein (arrow)) of the upper abdomen are visible. (**b**) Multiple round hypoechoic structures are visible (arrow). These represent pathologically enlarged lymph nodes close to the aorta (A).

Probe position 1b: abdominal lymph nodes

a. *Indication*: Disseminated abdominal TB should be considered in all HIV-positive patients with fever, unexplained loss of weight, weakness, diarrhea, and/or abdominal pain. Patients occasionally complain of longstanding hiccups. Abdominal masses and ascites may be found during clinical examination. The patients often show advanced immunosuppression with very low CD4 counts.

b. *Probe position*: The transducer is tilted back to a position more or less perpendicular to the patient's skin. In this position, the upper abdominal/periportal area can be visualized. The transducer is then slowly moved caudally to assess the periaortic area. To minimize the distance between the probe and the retroabdominal areas assessed, the abdominal wall should still be relaxed and the arms placed beside the abdomen.

c. *Normal finding*: Many physiological structures can be seen which are beyond the scope of the FASH examination. The examiner should attempt to locate the abdominal aorta as a landmark structure (Figure 3a). Once found, this structure should be followed caudally for approximately 10 to 15 cm.

d. *Pathological findings*: Lymph nodes larger than 1.5 to 2 cm could be considered pathologic in an HIV-infected individual [6]. Lymph nodes are usually hypoechoic (dark) round structures which appear in the US image (Figure 3b), increase in size as the transducer moves towards the center of the nodes, and then grow smaller again as the opposite site is reached. This phenomenon of ‘growing’ and ‘shrinking’ as the probe passes the node is easily recognized and gives the lymph nodes a blinking appearance (Additional file 3: Video 3). The node is measured at its maximal dimension.

e. *Interpretation*: Enlarged abdominal lymph nodes in a HIV-positive African patient with low CD4 counts are suggestive of abdominal TB. A chest X-ray should be done as the majority of patients with abdominal TB also have pulmonary changes suggestive of the disease [6]. If TB prevalence in the patient population is high, we would recommend starting TB treatment on clinical and imaging data. We recommend attempting to perform a follow up US after 6 to 8 weeks. In settings with lower prevalence and in cases of persistent lymphadenopathy, US-guided aspiration should be attempted. The aspirated lymph node material is sent for acid-fast stain (which is frequently positive), TB culture, and histology. In cases of persistent lymph nodes despite TB treatment, multi-drug resistant TB needs to be considered as well as other causes of persistent lymphadenopathy (especially lymphoma, KS, and non-TB mycobacterial infection). Non-compliance in taking the medication should be evaluated. It has to be noted that patients receiving TB treatment, and in particular when receiving concomitant ART, may show a initial increase of the size of the nodes due to immune reconstitution inflammatory syndrome [16]. This should not result in cessation of ART as it usually subsides during continued treatment.

**Figure 4:**
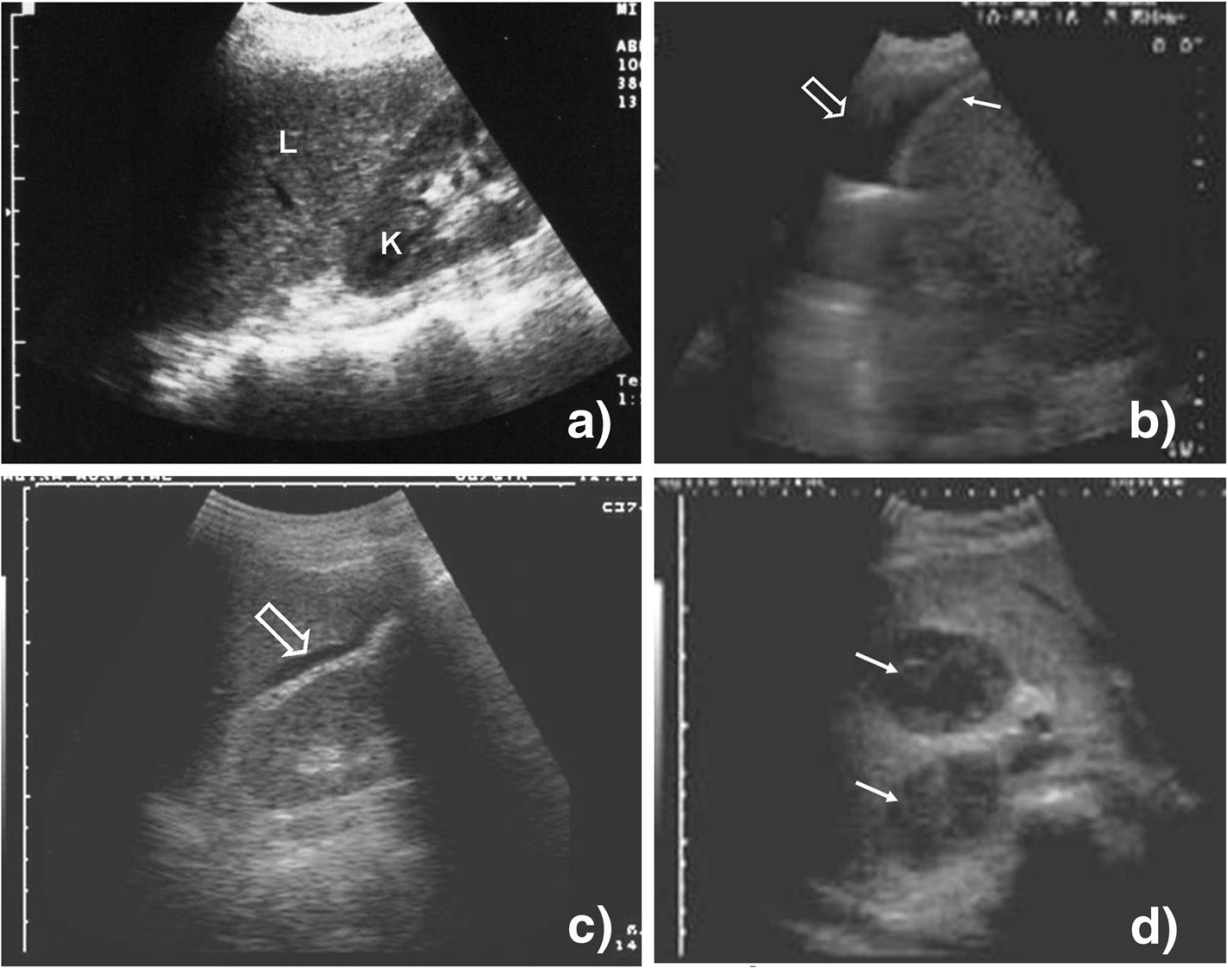
**Probe position 2, 3a, and 3b.** (**a**) Liver (L) and right kidney (K) are visible; there is no echo-free fluid above or below the liver. (**b**) An anechoic fluid collection is visible above the liver and the echogenic diaphragm (filled arrow) representing pleural effusion (open arrow) on the right side. (**c**) A small anechoic fluid collection is visible between the liver and the right kidney (open arrow) (Morrison's pouch). Free abdominal fluid can be diagnosed. (**d**) Two hypoechoic large lesions (filled arrow) can be seen in the parenchyma of the liver.

Probe position 2: pleural effusion right side

a. *Indication*: Pleural effusion should be considered in an HIV patient with pleuritic pain, irritating cough, or breathlessness. Dullness to percussion and reduced sounds of air entry are suggestive clinical findings; a chest X-ray might show opacity and displacement of the mediastinum to the contralateral side. Pleuritic TB may also be seen in patients with milder immune-suppression and even normal CD4 counts.

b. *Probe position*: The patient is asked to put his arms behind the head to free access to the side of the body. Additionally, this position widens the space between individual ribs. The transducer is positioned dorsal of the right mid-axillary line at the caudal part of the thorax. The transducer has to be adjusted so that the long axis is parallel to the ribs, allowing a transcostal view. It is important to place the transducer as dorsal as possible (close to the examination couch), as fluid collects in the dependant parts due to gravity.

c. *Normal finding*: The diaphragm and apical parts of the liver should be visible. If no effusion is present, the air in the basal parts of the lung will cause artifacts resembling a curtain that moves up and down with the respiratory cycle.

d. *Pathological findings*: Anechoic, black fluid may be visible in the costophrenic angle (Figure 4b). This is a pleural effusion, which is normally completely echo free but may contain internal echoes such as strands or smoke which are due to fibrinous structures or cells within the effusion (Additional file 4: Video 4).

e. *Interpretation*: A pleural effusion in HIV-positive patients is very suggestive of TB, especially when unilateral. Possible differential diagnoses of pleural effusions, particularly in bilateral effusions are generalized KS and other malignancies. KS often produces bloody or serosanguinous fluid when aspirated upon thoracentesis. Parapneumonic effusions should be included in the differential diagnosis. Patients usually have a high fever, show infiltrates on chest-X-ray along with a leukocytosis. Congestive heart failure needs to be considered as a reason for pleural effusion which would be supported by the sonographic demonstration of reduced left ventricular contraction.

Ultrasound can be used to guide thoracocentesis, and the fluid should be examined for TB. As in pericardial fluid, the AFB smear is often negative, but higher rates of positivity were seen in patients with low CD4 counts (<200 × 10^6^/L) [17]. TB culture is recommended but does not help a timely diagnosis. Data on newer methods like GeneXpert MTB are still sparse. In one study examining 113 pleural fluid samples, a specificity of 98% was reported, but sensitivity could not be calculated due to the low number of positive samples [15]. Until further data becomes available, we would recommend starting anti-TB treatment on clinical suspicion if other differential diagnoses above are unlikely.

Probe position 3a: ascites in the hepato-renal pouch (Morrison's pouch)

a. *Indication*: see probe position 1b.

b. *Probe position*: The transducer is moved a few centimeters caudally and is slightly rotated so that the longitudinal axis is parallel to the long axis of the body. Again, it is important to bring the scanner dorsally to see fluid in the dependent parts of the abdominal cavity.

c. *Normal finding*: The caudal edge of the liver and the right kidney should be visible. Between the two organs, there is usually an echogenic, white line (Figure 4a).

d. *Pathological findings*: If echo-free, black fluid is visible between the liver and the kidney (Figure 4c, Additional file 5: Video 5) or around the kidney. This is due to the free fluid in the abdominal cavity, i.e. ascites. Sometimes echogenic material like streaks or strands can be seen floating in the fluid, which might represent fibrin.

*e. Interpretation*: Ascites can be due to a number of reasons. In the HIV-infected patient, abdominal TB is one of the reasons, but other causes such as liver cirrhosis due to chronic hepatitis virus infection need to be considered. The finding of ascites needs to be interpreted in light of other clinical and laboratory findings. If possible, providers should attempt to assess sonographic signs of liver cirrhosis like a nodular surface, decreased vascular markings, and splenomegaly due to portal hypertension. These findings might be difficult to interpret for the less-experienced examiner. Ultrasound may be used to guide aspiration for lab investigation and culture.

Probe position 3b: focal liver lesions

a. *Indication*: see probe position 1b.

b. *Probe position*: The transducer is moved slightly up again to be positioned in the area of the liver. Again, the axis of the transducer needs to be adjusted to be parallel to the ribs to avoid artifacts from the bony structures of the thoracic cage.

c. *Normal finding*: The homogenous tissue of the liver is visible; inside, the hepatic vessels can be seen. The details of the liver anatomy lie beyond the FASH examination.

d. *Pathological findings*: Focal hypoechoic lesions might be seen and are relatively easy to recognize if they are large (Figure 4d).

e. *Interpretation*: In the tropical setting, hepatic abscesses are a common cause of abdominal complaints, especially in immunocompromised patients. These might be due to TB; however, other causes, especially amebic and bacterial abscesses, need to be considered, and in particular amebic abscesses are seen frequently (Additional file 6: Video 6). We recommend US-guided aspiration of the lesion; if the aspirate has ‘anchovy paste’ appearance, anti-amebic treatment is warranted [18]. AFB and Gram stain, as well as culture, should be done if possible.

**Figure 5:**
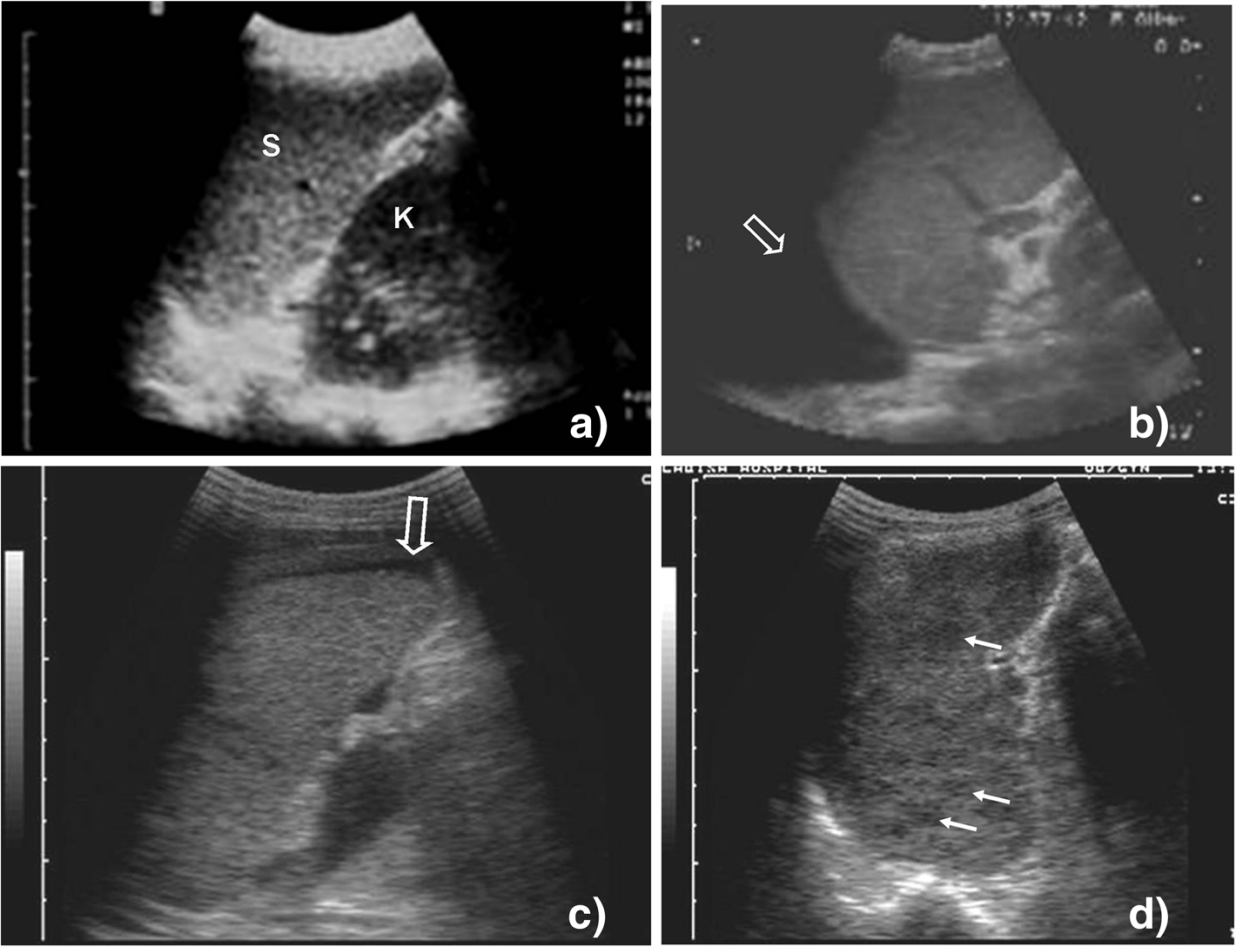
**Probe position 4, 5a, and 5b.** (**a**) Spleen (S) and left kidney (K) are visible; there is no echo-free fluid above or below the spleen. (**b**) An anechoic fluid collection is visible above the spleen representing pleural effusion (open arrow) on the left side. (**c**) An anechoic fluid collection is visible around the lower pole of the spleen (open arrow). Free abdominal fluid can be diagnosed. (**d**) Hypoechoic lesions can be seen inside the spleen (filled arrow). Micro-abscesses due to disseminated TB are a probable explanation.

Probe position 4: pleural effusion left side

a. *Indication*: see probe position 2.

b. *Probe position*: Mirroring the probe position 2, the transducer is placed on the left side of the thorax to assess for left pleural effusions.

c. *Normal finding*: The spleen and the left diaphragm should be visible; otherwise, the image is similar to 2.

d. *Pathological findings*: see probe position 2 (Figure 5b).

e. *Interpretation*: see probe position 2

Probe position 5a: ascites in the spleno-renal pouch

a. *Indication:* see probe position 1b.

b. *Probe position*: Mirroring the probe position 3a, the transducer is placed on the left flank to examine for free abdominal fluid in the dependent parts of the left abdominal cavity, i.e., between the spleen and kidney, and around the kidney. To achieve this, the transducer is moved caudally, and the long axis of the transducer needs to be more or less parallel to the long axis of the patient's body.

c. *Normal finding*: The spleen and the left kidney should be visible to ensure correct probe position (Figure 5a). Both organs should be separated by an echogenic, white line representing the capsules of the organs.

d. *Pathological findings*: Analogous to the hepato-renal pouch on the right side of the abdomen, free fluid might collect in the spleno-renal pouch and is visible as an anechoic, black fluid (Figure 5c).

e. *Interpretation*: Free fluid in the spleno-renal pouch represents ascites. As mentioned above, other causes of ascites should to be considered beside abdominal TB (see probe position 3a).

Probe position 5b: focal splenic lesions

a. *Indication*: see probe position 1b.

b. *Probe position*: Mirroring the probe position 3b, the transducer is moved slightly upwards and turned to be parallel to the ribs using the intercostal space as a window.

c. *Normal finding*: The homogenous tissue of the spleen should be visible.

d. *Pathological findings*: Hypoechoic, dark lesions in the spleen (size approximately 0.5 to 2 cm) (Figure 5d, Additional file 7: Video 7)

e. *Interpretation*: Multiple hypoechoic lesions may represent small abscesses, which are frequently secondary to disseminated TB [19]. The image is characteristic and other differential diagnoses like other infections or disseminated malignancy are less likely. As mentioned previously, diagnostic steps for concomitant pulmonary TB (sputum, chest X-ray) should be done. In a HIV-positive patient with low CD4 counts, we would have a low threshold to start anti-TB treatment on clinical grounds.

**Figure 6:**
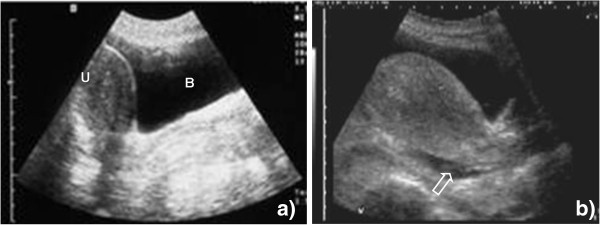
**Probe position 6.** (**a**) The pear-shaped uterus (U) is visible behind the fluid-filled bladder (B). There are no extra-vesical fluid collections, especially no collections in the Douglas' pouch behind the uterus. (**b**) Small, anechoic collection behind the uterus (open arrow), free abdominal fluid can be diagnosed.

Probe position 6: ascites in the pouch of Douglas

a. *Indication*: see probe position 1b.

b. *Probe position*: The probe is placed on the lower abdomen touching the upper rim of the symphysis pubis. The pelvic region can be scanned in the longitudinal axis (long axis of the scanner parallel to the long axis of the patient) or in the transverse axis (long axis of the scanner parallel to the upper rim of the pelvic bone). For visualization of parts deeper in the pelvis, the transducer has to be tilted upwards so that the caudal structures of the small pelvis become visible.

c. *Normal finding*: The bladder should be visible and its size will vary depending on how full it is. As urine is a fluid, it will be visible as an echo-free, black area, and care has to be taken not to confuse this with free fluid. Behind the bladder, the uterus might be visible as a pear-shaped organ in the female patient. Behind these structures and in front of the rectum, which cannot be seen due to air artifacts, the pouch of Douglas is located (Figure 6a).

d. *Pathological findings*: Echo-free, black areas might be seen behind the bladder (male patient) or behind the uterus (female patient) in the Douglas' pouch. These represent free fluid (Figure 6b, Additional file 8: Video 8).

e. *Interpretation*: Free fluid in the Douglas' pouch represents ascites. As mentioned above, other causes of ascites need to be considered beside abdominal TB. Very small amounts of fluid in the pelvis of the female patient may be normal and due to ovulation. In the female patient, pregnancies with amniotic fluid and ovarian cysts (filled with fluid) need to be considered. In comparison to free fluid, these differ in shape, are localized, and are usually limited by a wall.

## Conclusions

Focused assessment with sonography for HIV/TB is a technique that can be taught rapidly to physicians with little or no prior ultrasound experience. In the high prevalence setting, the learning process is facilitated by the fact that pathologic findings are present in a large proportion of HIV/TB co-infected hospital inpatients. The fact that many patients under investigation are underweight makes scanning easier because fat interfering with the scanning becomes less relevant. The examination takes only a few minutes and may provide important findings that alter patient management [10].

If available, a more comprehensive US scan of the abdomen of the HIV-infected patients may reveal other pathological findings [20] such as HIV-associated cardiomegaly or findings of HIV nephropathy. Although these are important, they are not part of the routine FASH as they are more difficult to recognize or lack direct therapeutic interventions.

It is important to remember that the FASH examination results have to be interpreted by a clinician within the clinical and epidemiological framework applying to the individual patient. In patients with findings suggestive of EPTB, a sputum examination [21] and a chest X-ray [6] should be performed as these frequently show additional abnormal results. The protocol is intended for settings where the prevalence of HIV/TB co-infected patients is high as in many hospitals in Southern and Eastern Africa. Additionally, TB wards or infectious disease departments in West Africa, Asia, or South America may benefit from the protocol as here the prevalence of HIV/TB is increased because pre-selected, referred patients are seen. In these settings, there is a high prevalence (pre-test probability) of HIV and TB; even in an exam in which findings are only suggestive (non-ideal sensitivity and specificity) of TB, these findings may be enough to predict the presence of the disease (post-test probability). In South Africa, the Emergency Medicine Association adopted FASH as a module in their US training curriculum and uses it in the emergency-room setting. It has recently been shown as one of the most frequently used modules in their ultrasound repertoire [22].

FASH does not provide the health care worker with a definite diagnosis but may reveal findings that point in the direction of EPTB. The potential public health benefit of the protocol may be far reaching, in terms of more rapid identification of EPTB cases even at a peripheral hospital level, where imaging modalities are scarce and most HIV and TB care will be delivered in the future.

## Competing interests

The authors declare that they have no competing interests.

## Authors’ contributions

TH developed the FASH protocol and used it extensively during ultrasound teaching, and assisted in drafting the manuscript. CW drafted the manuscript. SG revised the manuscript and added clinical discussion. EB used the FASH protocol during the ultrasound teaching and revised the clinical discussion. All authors read and approved the final manuscript.

## Authors’ information

TH is an internal medicine and infectious disease physician with long experience in teaching ultrasound. He developed the FASH protocol in Hlabisa Hospital, a district hospital in rural KwaZulu-Natal, South Africa. He taught and teaches FASH ultrasound in Pavia, Italy as well as in many African countries. CW is a microbiologist, infection control specialist, and infectious disease clinician with a strong interest in ultrasound, diagnostic methods, and epidemiology of HIV and TB. SG is an internal medicine-pediatric trained physician with an interest in research and clinical teaching of ultrasound in resource limited settings. EB is an infectious diseases clinician with long term experience in ultrasound and an assistant professor of infectious disease at the University of Pavia. He is the director of the ‘Short Course on Abdominal Ultrasound in Infectious and Tropical Diseases’ (http://www.tropicalultrasound.org) held annually in Pavia, Italy, and the co-director of the course ‘Clinical Ultrasound in Tropical Infectious Diseases’ held annually in Lima, Peru. Both courses are co-sponsored by the World Health Organization, the American Society of Tropical Medicine and Hygiene, and WINFOCUS.

## Supplementary Material

Additional file 1 **Pericardial effusion.** Note fibrinous material on the epicardial side. Click here for file

Additional file 2 **Pericardial tamponade.** Tamponade with impaired filling of the right ventricle. Note the changes of the size of the right ventricle during respiratory cycle. Click here for file

Additional file 3 **Abdominal lymph nodes.** The lymph nodes next to the liver are enlarged and have a hypoechoic appearance. Click here for file

Additional file 4 **Pleural effusion.** Scan of the left side. The spleen is briefly visible; there are fibrin strands floating in the effusion. Click here for file

Additional file 5 **Ascites in Morrison's pouch.** This can be seen as anechoic area between liver and kidney. Click here for file

Additional file 6 **Liver abscess (amebic).** A large (approximately 8 cm) homogenous lesion is seen in the parenchyma of the liver. By aspiration, an amebic liver abscess was diagnosed. Click here for file

Additional file 7 **Multiple hypoechoic focal lesions.** The lesions are visible in the spleen representing micro-abscesses due to disseminated TB. Click here for file

Additional file 8 **Ascites in the pouch of Douglas.** Anechoic fluid can be seen behind the bladder and uterus. Click here for file
